# The effect of smoking on lung cancer: ethnic differences and the smoking paradox

**DOI:** 10.4178/epih.e2016060

**Published:** 2016-12-20

**Authors:** Keum Ji Jung, Christina Jeon, Sun Ha Jee

**Affiliations:** 1 Institute for Health Promotion, Department of Epidemiology and Health Promotion, Graduate School of Public Health, Yonsei University, Seoul, Korea; 2 Department of Public Health, Graduate School, Yonsei University, Seoul, Korea

**Keywords:** Smoking, Lung neoplasms, Risk, Ethnic groups

## Abstract

The objectives of this review were to determine whether the smoking paradox still exists and to summarize possible explanations for the smoking paradox. Based on published data, we compared the risk of cigarette smoking for lung cancer in Western and Asian countries. We extracted data from the relevant studies about annual tobacco consumption, lung cancer mortality rates according to smoking status from each country, and possible explanations for the smoking paradox. A significantly greater risk of lung cancer death was found among current smokers in Asian countries than among nonsmokers, with relative risks (RRs) of 4.0 to 4.6 for Koreans, 3.7 to 5.1 for Japanese, and 2.4 to 6.5 for Chinese. Although a significantly greater risk of lung cancer was present among current smokers in Asian countries, the RRs in Asian countries were much lower than those reported in Western countries (range, 9.4 to 23.2). Possible explanations for the smoking paradox included epidemiologic characteristics, such as the smoking amount, age at smoking initiation, and the use of filtered or mild tobacco. The smoking paradox definitely exists, but may be explained by major epidemiologic characteristics. Therefore, the smoking paradox should not be interpreted as indicating that tobacco is safer or less harmful for Asians.

## INTRODUCTION

Tobacco has a long history of use starting in the early Americas. Following the discovery of tobacco in the New World by Christopher Columbus in 1492 [1,2], it was introduced to European countries. Cigarettes became popular after the Industrial Revolution. Tobacco was popularized in two especially significant periods. The first wave of popularization occurred in Western countries at the time of World War I, which started in 1914. The second wave was in Asian countries around the 1940s, an epidemic that started 40 years after the European countries. Popularization continued until scientific studies in the mid-20th century demonstrated the negative health effects of tobacco smoking, including lung cancer.

Despite the lack of data for these periods, the prevalence of smoking is thought to have increased during World Wars I and II. In 1935, corresponding to the development of a machine by a British company, the prevalence of smoking increased. The tobacco industry requires mass production of cigarettes. The first epidemiological paper on this topic published in the first half of the 20th century stated that “smoking is connected with the shortening of life span,” and smokers began to reflect on their health because they observed some negative health effects of smoking [3]. In 1950, cigarette companies initiated a shift in cigarette design from a non-filter design to a filter design. Production of filter cigarettes skyrocketed from 0.5% in 1950 to 87.7% in 1975 [4].

**Figure 1.**
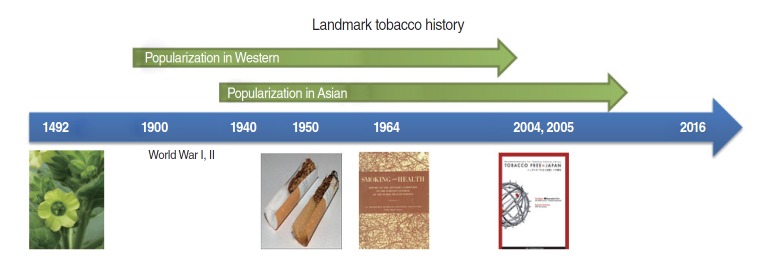
Landmarks of the history of tobacco, from discovery to popularization.

In 1950, Doll & Hill [5] conducted the British Doctors’ Study to determine the association between smoking and health consequences. In 1964, the first Surgeon General’s Report was published in the US, which allowed governmental enforcement of smoking bans in public places and strongly recommended smoking cessation [6]. Forty years later, another comprehensive report entitled Tobacco Free ^*^ Japan was published in 2004 in Japan [7]. At that time, findings on smoking and cancer risk obtained using data from the Korean Cancer Prevention Study (KCPS) were also published [8]. Interestingly, however, the magnitude of the effect of smoking on lung cancer was different between Western and Asian countries, with a smaller effect size observed in Asian countries. Investigators named this phenomenon “the smoking paradox,” and have tried to explain it [9,10]. The objectives of this review were to investigate whether the smoking paradox still exists and to summarize possible explanations for the smoking paradox (Figure 1).

## DOES THE SMOKING PARADOX EXIST?

To determine whether the risk of smoking for lung cancer is lower in Asian countries, which have been referred to as the smoking paradox, we reviewed selected well-established epidemiologic studies from Western and Asian countries. The results are presented below.

### United Kingdom

The British Doctors’ Study is the oldest epidemiologic study on this topic from the United Kingdom (UK) and has become very well known. This UK prospective study of smoking and death began in 1950, included 34,439 British male doctors, and has now continued for 50 years. The researchers published a 20-year follow-up study in 1976, a 40-year follow-up in 1994, and a 50-year follow-up in 2004 [11-13]. Recently, results from a 60-year follow-up study were presented at a meeting in honor of Richard Doll’s centenary at Oxford University on October 28, 2012.

**Figure 2.**
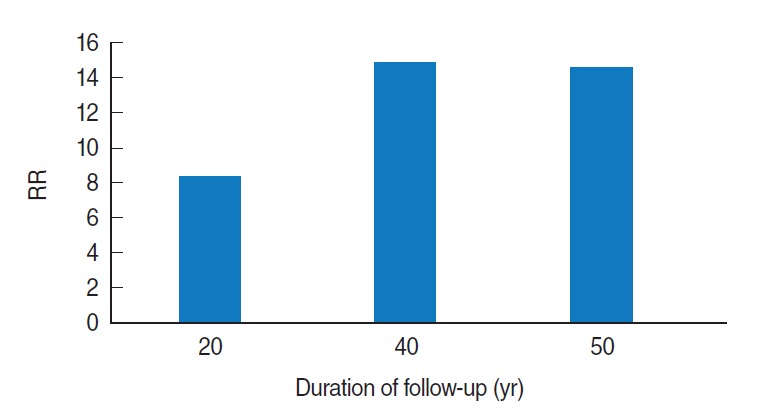
Relative risk (RR) of smoking for lung cancer, UK. Adapted from Doll et al. [11-13].

In the latest three reports (the 20-year, 40-year, and 50-year follow-up) the relative risk (RR) of smoking for lung cancer was found to have increased over the duration of follow-up. The RR was approximately eight at the time of the 20-year follow-up study, but increased to above 14 when the participants were followed up for more than 40 years (Figure 2).

### US

In the US, the most well-known prospective cohort that we reviewed was the US veterans study [14]. The tobacco use of almost 250,000 US veterans was surveyed via questionnaire in the 1950s. Together with six other large studies and several case-control studies, the early results of this investigation provided the basis for the first series of reports by the Surgeon General on the health effects of smoking, which identified lung cancer and a number of other smoking-related diseases [6]. In a graph showing the results from the US veterans study cohort published in 1995, the RRs of smoking for laryngeal cancer and lung cancer were 17.8 and 11.7, respectively [14].

Additionally, the Cancer Prevention Study (CPS), the largest prospective cohort, was reviewed. In 1959, 1,078,894 subjects were recruited by the American Cancer Society to participate in the 12-year study, CPS-I. CPS-II, a prospective mortality study, was initiated by the American Cancer Society in 1982 [15]. The American Cancer Society recruited subjects in 1982 among the friends, neighbors, and acquaintances of volunteers. The tobacco use of almost more than 1.2 million US inhabitants was surveyed by the questionnaire in 1982. The key findings were remarkable. On average, the mortality among non-smokers was 8.6, while the mortality among smokers was remarkably high (101.3 per 100,000 persons). The estimated RR was 11.9. Smoking accounted for over 95% of lung cancer deaths in men and 92% in women.

### China

China is by far the largest producer of cigarettes in the world. Between 1915 and 1945, annual cigarette consumption rose from approximately 0 to 100 billion. China is also the largest consumer market in the world, with over 300 million smokers consuming 1.7 trillion cigarettes in 1997 (unpublished data). Three prospective studies of smoking and the RR for lung cancer have been conducted: the Shanghai cohort, whose sample size was 18,244, and two prospective cohorts studied by Lam et al. [16] and Yuan et al. [17]. In the Shanghai cohort, male residents of Shanghai, China were enrolled from January 1, 1986, through September 30, 1989 and were actively followed via annual visits during the study period [17]. However, the cohort size was small and the duration of follow-up was relatively short. In China, Liu and Peto published a paper [18] presenting a retrospective proportional mortality study of one million deaths in 1998. The goal of that study was to examine the hazards at an early phase of the growing epidemic of death from tobacco in China. From 1989 to 1991, they interviewed the surviving family members of one million people who died from 1986 to 1998 in 98 areas of China. For both sexes, the lung cancer rates for individuals 35 to 69 of age were approximately 2.6 times higher in smokers than in nonsmokers among men and 2.0 times greater in smokers than in non-smokers in women. Of all deaths attributed to tobacco, 45% were due to chronic obstructive pulmonary disease and 15% were due to lung cancer.

### Japan

In 2004, Tobacco Free * Japan, a comprehensive and detailed report on smoking and health similar to the US Surgeon General’s report was published in Japan. Although the report covered numerous epidemiologic studies, we selected the Japan Collaborative Cohort (JACC) study for this review [19]. The JACC study was sponsored by the Ministry of Education, Culture, Sports, Science, and Technology of Japan. The cohort was established from 1988 to 1990, with 46,465 men and 64,327 women aged 40 to 79 years recruited from 45 study areas throughout Japan. The RRs for lung cancer in smokers were 4.46 in men and 3.58 in women. In men, the population-attributable risk (PAR) was 52.2% for current smokers and 14.8% for ex-smokers.

### Korea

**Figure 3.**
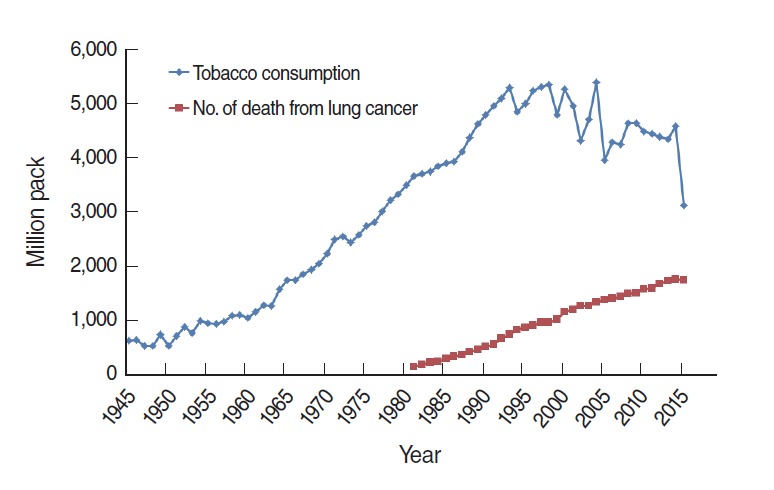
Tobacco consumption and lung cancer deaths in Korea.

In Korea, despite the introduction of tobacco 400 years ago, its widespread use in the form of manufactured cigarettes only emerged after 1945. The first national brand, produced by a government monopoly, was named *Seung Ri*, meaning “independence from Japan.” Cigarette consumption rose sharply after the end of the Korean War in 1953. The rise has been particularly pronounced since 1960, as Korea became economically prosperous, with a peak around 2000 (Figure 3) [20]. Korean tobacco consumption trends have shown an increase since 1945. In Figure 3, the tobacco consumption amount unit is one million packs per year, and the red line shows the number of deaths from lung cancer. Since 1981, the year of the first mortality report, the number of lung cancer deaths in Korea continuously increased until 2015, when a slight drop was observed for the first time. The KCPS is a prospective cohort study with a follow-up period of 24 years that was designed to assess the risk factors for mortality, incidence, and hospital admission due to cancer and other chronic diseases. The KCPS cohort included 1,329,525 Koreans from 30 to 88 years of age who underwent biennial medical evaluations from 1992 to 1995. The first report based on the KCPS was published in 2004. The RRs of smoking for lung cancer were 4.6 in men and 2.5 in women [20].

### Summary of selected publications

Two major observations were made based on our analysis of the data. First, a significantly greater risk of lung cancer mortality was found among current smokers than among non-smokers in Asian countries (RRs, 4.0 to 4.6 for Koreans; 3.7 to 5.1 for Japanese; and 2.4 to 6.5 for Chinese). Second, although a significantly increased risk of lung cancer was present among current smokers in Asian countries, the RRs in Asian countries were much lower than those reported in Western countries (range, 9.4 to 23.2). The results were very similar among men, with the RRs in Asian countries being much lower than those reported in Western countries. Asian countries had a lower PAR for smoking and lung cancer than Western countries. In summary, numerous epidemiological studies have consistently reported smoking to be a risk factor for lung cancer. However, the magnitude of the risk for lung cancer mortality associated with cigarette smoking has been reported to be lower in Asian countries than in Western countries.

## POSSIBLE EXPLANATIONS FOR THE SMOKING PARADOX

### Different epidemics

Cigarette consumption in Japan and Korea has followed a pattern similar to that observed among adults in the US, although the major increase took place 40 years later [7]. One of the possible explanations for this discrepancy may be the differences in the exposure amount of cigarettes smoked in different eras in Western countries and Asian countries. The per capita cigarette consumption in the US reached 3,000 cigarettes per year in the 1940s, while Korea and Japan reached the same level in the 1970s. Therefore, we may hypothesize that Asian countries have not yet experienced the full effect of cigarette smoking on lung cancer.

### Age at smoking initiation and amount of smoking

Another explanation might be the age of smoking initiation. It has been found that current smokers in Asian countries began smoking at an older age. A strong trend has been found for an earlier smoking initiation age in later birth cohorts. While the age at smoking initiation for Korean men and women has gradually decreased in recent birth cohorts, they still began smoking much later than men and women in the US [20]. The average smoking amount among Asian smokers was relatively small in the past. In China, the average daily cigarette consumption for men was one in 1952, increasing to 16 in 1996 [18]. In Korea, the weighted mean of smoking quantity among current smokers was only a half-pack in 1980, which dramatically increased to almost one pack in 2000 (unpublished data).

### Toxicity and filter

The contents of tobacco itself have not significantly changed over time. Basic physiology and genetic factors are likewise not changeable over time. However, tobacco products may have changed over time. Since consumer demand has increased for mild tobacco due to health concerns, tobacco industries have produced products such as Mild Seven or Right, which are known to have low tar and low nicotine. Recently, such brands have become very popular in Asian countries. Previous studies have found that the use of low-nicotine products did not lead smokers to change the quantity of nicotine consumption per day. This is because smokers who use low-nicotine products tend to increase the number of cigarettes they smoke to meet their needs for nicotine. Therefore, the effects of low-tar or low-nicotine product use on various diseases are known to be similar to those of standard cigarettes.

However, Philip Morris, British American Tobacco, and other tobacco companies considered the possibility of reducing cigarette toxicity through the use of filters. Although filtered brands constitute over 90% of the entire cigarette market [4], epidemiologic data support the conclusion that cigarette filters have done little to protect smokers. In 2011, investigators from the US and Japan reported that the shift from non-filter to filter cigarettes appears to have merely altered the most frequent type of lung cancer, from squamous cell carcinoma to adenocarcinoma [21].

### Lung cancer risk of never smokers

**Figure 4.**
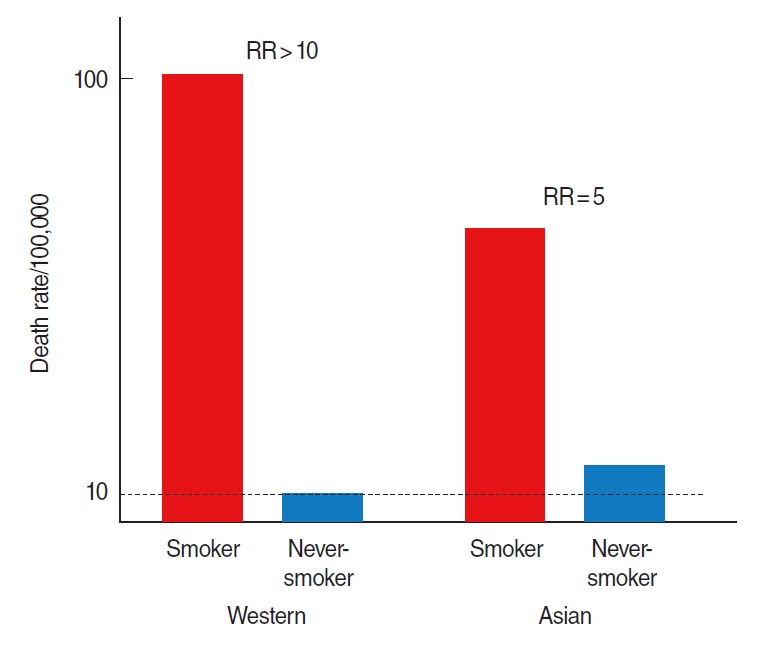
Hypothetical graph of relative risk (RR) in relation to death rate and smoking status.

The data previously discussed in this review indicate that lung cancer mortality among nonsmokers across countries varies, and is likely to be lower in Western countries than in Asian countries. For men, lung cancer mortality per 100,000 persons among non-smokers in Western countries ranged from 14 to 17, while in Asians it ranged from 19.2 to 36.0. Death rates of non-smokers in all Asian cohorts were higher than those of non-smokers in Western countries. However, the death rates for current smokers in all Asian cohorts were lower than those in Western countries. Comparing the death rates and exposure levels of current smokers indicates that one reason for the lower RR in Asians is the presence of higher death rates among non-smokers combined with lower death rates among smokers (Figure 4). Another comprehensive study reported that the death rate from lung cancer among never smokers was higher in men than in women, and higher in Asians residing in Asia than in individuals of European descent [22]. This study used participants from the CPS to identify mortality rates among those of European descent and participants from the KCPS as the Asian sample. The morality rates per 100,000 persons among Europeans and Asians were 12.0 and 26.0 in men and 9.5 and 16.1 in women, respectively. One of the possible reasons for the increased lung cancer risk among never smokers in Asian countries is poor ventilation combined with the use of coal, which Asian women have traditionally been exposed to [23]. Genetic risk scores involving telomere length are also a possible factor explaining differences in lung cancer risk among never smokers [24].

### Genetic differences

The association of DNA methylation with smoking has been reported to vary according among ethnic groups [25]. Current smokers in Europe have been found to smoke more per day, to have started smoking earlier, and, therefore, to have accumulated more pack-years than South Asians. Differences were found in DNA methylation between South Asians (57%) and Europeans (53%) among current smokers. However, the difference was not sufficiently large and may not explain the smoking paradox. Another issue is epidermal growth factor receptor (EGFR). The *EGFR* gene is a major oncogene in lung cancer adenocarcinoma in Asians. The expression of EGFR in both never smokers and ever smokers was found to be much higher in Japan than in the US [26]. These differences may explain the smoking paradox, at least for lung cancer adenocarcinoma in Asians.

### Others

In summary, smoking is the most convincing causal risk factor for lung cancer, with over 90% of attributable risk in Western countries. Alcohol consumption, cholesterol, total and saturated fat, and animal fat are other possible risk factors. Vegetables and fruits are convincing protective factors, while physical activity, carotenoids, vitamin C, and selenium are potentially beneficial. In a report that compared the outcomes of tobacco smoking (e.g., lung cancer) between Japan and the US, no differences were shown regarding diet, degree of inhalation, or alcohol consumption. However, more toxicity due to the carcinogens in cigarettes and an earlier onset age of smoking were found in the population in the US [27].

## CONCLUSION

Based on previous epidemiological studies, the smoking paradox definitely exists. However, while the smoking paradox may be explained by major epidemiologic differences between Western and Asian populations, such as smoking amount, age at smoking initiation, and the use of filtered or mild tobacco, we cannot conclude how much these factors may explain this phenomenon, as relevant genetic differences have not been sufficiently elucidated.

The smoking paradox itself bears important implications for public health. Based on previous studies that examined differences in epidemiological characteristics related to the smoking paradox, the Asian population may not yet have fully experienced the damage from smoking due to a comparably shorter tobacco epidemic history than Western countries. Second, the Asian population has been found to be characterized by a late age of smoking initiation, and since the smoking rate has increased and the initiation age of adolescents has decreased recently, the damage from smoking may increase in the future. In the public health field, practitioners should be especially cautious of misstating the smoking paradox by saying that tobacco is safer or less harmful for Asians. Although tobacco brands change over time, the contents of tobacco do not, meaning that no safe tobacco brand exists in the world.
